# The Future of “Omics” in Hypertension

**DOI:** 10.1016/j.cjca.2016.11.023

**Published:** 2017-05

**Authors:** Gemma Currie, Christian Delles

**Affiliations:** Institute of Cardiovascular and Medical Sciences, University of Glasgow, Scotland, United Kingdom

## Abstract

Despite decades of research and clinical practice, the pathogenesis of hypertension remains incompletely understood, and blood pressure is often suboptimally controlled. “Omics” technologies allow the description of a large number of molecular features and have the potential to identify new factors that contribute to blood pressure regulation and how they interact. In this review, we focus on the potential of genomics, transcriptomics, proteomics, and metabolomics and explore their roles in unraveling the pathophysiology and diagnosis of hypertension, the prediction of organ damage and treatment response, and monitoring treatment effect. Substantial progress has been made in the area of genomics, in which genome-wide association studies have identified > 50 blood pressure–related, single-nucleotide polymorphisms, and sequencing studies (especially in secondary forms of hypertension) have discovered novel regulatory pathways. In contrast, other omics technologies, despite their ability to provide detailed insights into the physiological state of an organism, have only more recently demonstrated their impact on hypertension research and clinical practice. The majority of current proteomic studies focus on organ damage resulting from hypertension and may have the potential to help us understand the link between blood pressure and organ failure but also serve as biomarkers for early detection of cerebrovascular or coronary disease. Examples include signatures for early detection of left ventricular dysfunction or albuminuria. Metabolomic studies have the potential to integrate environmental and intrinsic factors and are particularly suited to monitor the response to treatment. We discuss examples of omics studies in hypertension and explore the challenges related to these novel technologies.

## Résumé

La pathogenèse de l’hypertension demeure toujours partiellement incomprise en dépit de décennies de recherche et de pratique clinique, ce qui fait en sorte qu’on peine encore souvent à maîtriser de manière optimale la pression artérielle (PA) des patients. Les technologies « omiques » permettent de décrire un nombre important de caractéristiques moléculaires tout en fournissant la possibilité de cibler de nouveaux facteurs ayant une incidence sur la régulation de la PA et sur leurs interactions. Dans cet article, nous mettons l’accent sur le potentiel de la génomique, de la transcriptomique, de la protéomique et de la métabolomique ainsi que leur rôle dans l’élucidation de la physiopathologie et le diagnostic de l’hypertension, la prédiction des dommages organiques et de la réponse au traitement ainsi que la surveillance des effets du traitement de l’hypertension. D’importants progrès ont été réalisés grâce à la génomique puisque les études d’association pangénomiques ont permis jusqu’ici d’identifier plus d’une cinquantaine de polymorphismes de nucléotides individuels ayant une incidence sur la pression artérielle et que les études de séquençage (particulièrement en ce qui a trait aux formes secondaires d’hypertension) ont permis de mettre au jour de nouvelles voies de régulation. Les autres technologies « omiques », en dépit de leur capacité à fournir un portrait détaillé de l’état physiologique d’un organisme, n’ont que tout récemment laisser entrevoir leur utilité en matière de recherche et de pratique clinique relativement à l’hypertension. À l’heure actuelle, les études protéomiques se concentrent sur les dommages organiques causés par l’hypertension, ce qui pourrait nous aider à élucider le lien entre la pression artérielle et l’insuffisance organique, en plus de nous fournir des biomarqueurs permettant le dépistage précoce de la maladie vasculaire cérébrale ou coronarienne. À titre d’exemple, on peut citer les facteurs permettant le dépistage précoce de la dysfonction ventriculaire gauche ou de l’albuminurie. Les études métabolomiques, pour leur part, permettent de tenir compte des facteurs environnementaux et intrinsèques, ce qui signifie qu’elles sont particulièrement adaptées pour la surveillance de la réponse au traitement. Nous traitons de diverses études « omiques » relatives à l’hypertension et nous examinons les défis posés par ces technologies novatrices.

Hypertension is a condition that is difficult to define. Over the years, guideline committees and scientific societies have suggested definitions that are based on blood pressure values, long-term risk, and treatment effects. A recent Lancet Commission report pragmatically categorizes individuals as hypertensive “when they persistently cross the blood pressure threshold above which there is robust scientific evidence that antihypertensive treatment will improve their prognosis.” This definition is based on the finding that blood pressure levels, at least within a wide range, are continuously related to cardiovascular risk and that there is no biologically plausible blood pressure threshold at which normal physiology becomes a pathologic process.^1^ The challenges in the definition of hypertension are important in the context of this review and in the wider context of precision medicine in this series of articles in the *Canadian Journal of Cardiology*, in which precise molecular biology methods are applied to a condition that is inconsistently defined.

In this article, we explore how “omics” technologies can further our understanding of the pathophysiology of hypertension and influence patient management. We focus on specific omics technologies and on human studies but appreciate that a wealth of data has been generated in rodent and other experimental models.

## The Role of Omics Technologies in Cardiovascular Diseases

Cardiovascular diseases generally result from interaction between genetic factors and environmental influences. The relative contribution of these components to disease phenotype varies. For example, blood pressure in patients with monogenic forms of hypertension is driven mainly by genetics but can still be modified by environmental factors such as salt intake and diuretic use. In contrast, essential hypertension often manifests in concert with conditions such as obesity and insulin resistance, and blood pressure responds at least to some extent to lifestyle modifications; despite this, essential hypertension exhibits a high degree of heritability.^2^, ^3^ If all genetic and environmental factors could be captured comprehensively in an individual throughout life, it should theoretically be possible to precisely map present disease status, future disease progression, and long-term complications. Indeed, recent advances in genotyping technologies, such as next-generation sequencing, have helped address at least the first part of the equation. Even if our understanding of the complexity of the human genome, tools to analyze large-scale genetic data, and knowledge of the links between genetic variation and pathophysiology of disease are still very limited, one can at least imagine that a meaningful description of an individual's genome is possible. In contrast, a comprehensive description of all the environmental factors an individual has ever been exposed to is by far more challenging and probably impossible. As a result, the second part of the equation remains unknown and can be approximated only by gathering information on smoking status, physical activity, social deprivation, salt intake, and other environmental factors. More comprehensive assessment of phenotypes is an important principle of precision medicine, in which therapeutic approaches are tailored to the exact condition of the patient. In fact, the term “phenomics” has been coined based on the “phenome” that describes the material basis of the phenotype.^4^

It is this precise characterization of the genotype and phenotype that can be achieved with omics technologies—both in isolation and combined with other tools, including imaging techniques, functional testing, and the art of comprehensive history taking. We briefly describe the 4 main omics technologies that form the focus of this article before exploring their use in hypertension.

## Why Does Hypertension Need Omics?

With the paradigm of molecular biology in mind that DNA is transcribed to RNA and translated to proteins that then control metabolism, we focus on a comprehensive assessment of these 4 steps by the means of genomics, transcriptomics, proteomics, and metabolomics. Apart from these omics technologies, other types of omics play important roles in biomedical research: Epigenomics studies the range of modifications of DNA, eg, by describing genome-wide DNA methylation data; degradomics studies the breakdown of proteins by focusing on all proteases, their substrates, and their inhibitors; and lipidomics is a subtype of focusing on lipid classes and subclasses. We will not describe the relevant technical principles in great detail and instead refer the reader to Table 1 in which these principles are summarized.

**Figure 1:**
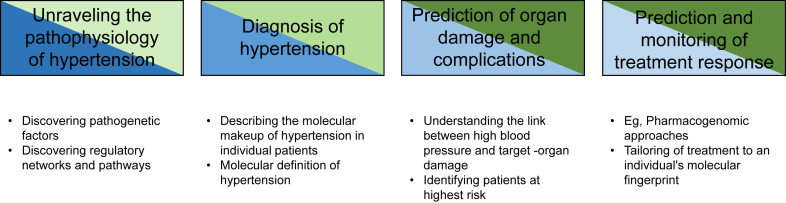
Applications of omics technologies in hypertensions research. Research and clinical needs are shaded blue and green, respectively. Darker colours indicate more urgent needs.

Omics technologies can significantly contribute to our understanding of the pathogenesis of hypertension, and this is indeed the area in which much progress has been made in recent years, especially in genomics. Equally exciting, there is potential for translation into clinical practice to aid in the diagnosis and management of patients with hypertension. The areas of research and clinical practice that are most likely to benefit from omics-based data are presented in Figure 1.

We outline further how these aims can be addressed in the future by omics technologies. Before we do this, we discuss the current state of omics in hypertension. Each individual omics approach has associated strengths and weaknesses, and therefore not all technologies are suited for all tasks. It is most important to recognize that precision medicine cannot rely on 1 technique in isolation; rather, they may be complementary to one another in characterizing disease processes.

## Present Status of Omics in Hypertension

In this section, we give an overview of the present work that has used genomics, transcriptomics, proteomics, and metabolomics to study various aspects of human hypertension. This is not a systematic review; rather, it highlights noteworthy achievements and represents the breadth of studies that have already been undertaken and the specific challenges related to them. There are, however, a number of general challenges that omics-based research has faced in recent years:

1. The definition of hypertension is not particularly precise and has changed over time. Of course, this affects all research into hypertension, but one should be aware of this limitation when expectations in the seemingly all-encompassing omics technologies meet a clinical phenotype that is loosely defined and, as stated by Kaplan in 1998, often is not measured accurately.^5^
2. Essential hypertension is a multifactorial disease, and to detect these factors, large sample sizes are required. With the reduced costs of genotyping, very large-scale genomic studies have been conducted recently, whereas costs for other omics approaches are still high, studies are labor-intensive, and available studies are by far not as large as those in genomics.
3. Hypertension is a disease in its own right but is tightly linked to its sequelae (eg, vascular stiffening) and to conditions that lead to hypertension (eg, renal diseases). Without knowledge of the presence or absence of other cardiovascular, renal, and metabolic phenotypes, it is difficult to prove a causal link between molecular features and hypertension and exclude their association with these other phenotypes. Large sample sizes with appropriate statistical adjustment, deep phenotyping, and experimental studies to dissect the mechanisms are required.^6^
4. The duration of the development of hypertension and its association with age is a challenge. Researchers shy away from long-term longitudinal studies in humans, and most omics studies are therefore cross-sectional in nature. In fact, literature searches for the terms “omics” and “hypertension” result in a relatively large number of articles on pulmonary hypertension and pre-eclampsia—conditions that develop much faster than essential hypertension.
5. The platforms used for omics research are complex and require specialist knowledge, and results from different platforms often are not comparable. Without standardization and better recognition of strengths and weaknesses of each approach, clinical translation will be challenging.
6. Although the somatic genome can be studied in virtually any DNA-containing material, the transcriptome, proteome, and metabolome are much more cell and tissue specific. Obtaining the right samples for the right clinical and research questions is therefore particularly important, and technical aspects of sample handling and conservation need to be observed.^7^

## Genomics

Genomics has probably seen the most dramatic development of all omics technologies in the past decade. The **Bri**tish **G**enetics of **H**yper**t**ension (BRIGHT) study that was published < 15 years ago still used microsatellite markers and linkage analysis to provide genome-wide information in 2010 sibling pairs.^8^ By 2007, the Wellcome Trust Case Control Consortium was able to analyze a total of 17,000 samples using chip-based technology to study approximately 500,000 variants across the genome.^9^ Further development in technology and reductions in costs have led to larger studies at even higher resolution.^10^ These studies have identified > 50 single-nucleotide polymorphisms robustly associated with hypertension or blood pressure, or both, and related to functional pathways such as signalling, renal function, and natriuresis.^11^

The vast majority of studies were conducted in cohorts that display a continuum of blood pressure. A notable exception is a study that used an extreme case-control design and was thereby able to detect a statistically significant signal in a considerably smaller sample size^12^; the robust association of a variant within the *UMOD* gene encoding the protein uromodulin in this study is also remarkable in that most other signals from genome-wide association studies (GWAS) are not located in or near genes but rather in intergenic regions, where an immediate functional role of these variants is more difficult to elucidate. In fact, only a few GWAS signals have been directly linked to a functional pathway, and each variant contributes to only minute changes in blood pressure in the range of 1 mm Hg systolic blood pressure or 0.5 mm Hg diastolic blood pressure.^10^

With further advanced technology, we are now in a position to sequence individual genes, all exomes, and even the whole genome quickly at relatively low cost. Results of a large exome-based study that discovered novel genetic loci associated with hypertension have been published recently, showing that rare variants can have larger effects on blood pressure (> 1.5 mm Hg) than common variants.^13^ However, sequencing techniques have also been used to identify variants in specific genes. Ji et al.^14^ used this approach to demonstrate that variants in genes encoding monogenic forms of hypertension are also present in essential hypertension and explain a significant proportion of blood pressure variability. In individual patients in whom clinical investigation indicates secondary hypertension, sequencing analysis has repeatedly identified new genetic variants and new mechanisms of blood pressure regulation, including *KCNJ5* mutations associated with adrenal hyperplasia and mutations of *CACNA1D* encoding a voltage-gated calcium channel causing cause adrenal aldosterone-producing adenomas in the absence of *KCNJ5* mutations.^15^, ^16^

There is no doubt that genomics has firmly established itself in hypertension research and has already generated a wealth of data providing new insights into the physiology and pathophysiology of blood pressure regulation. Technologies from chip-based genotyping arrays to next-generation sequencing are robust and precise, and further improvements can be expected. Most importantly, genomic studies can be performed in virtually any sample that contains DNA because the genetic information is the same or at least similar for the majority of the genome across all cells. In addition, the genome changes only minimally with advancing age, and any changes are mostly restricted to the telomeres and to rare de novo mutations, eg, in the development of tumours. Genomic analyses can therefore be performed at any age and are thus particularly suited to preclinical predictive studies in young individuals.

There are some general caveats, however, that should be considered. First, even if genes do not change with ageing, their expression patterns alter substantially. In childhood, a range of genetic variants are associated with hypertension in different age groups,^17^ and the interaction with age and environmental factors results in varied gene expression between individuals with the same genotype. Second, although the genetic sequence does not undergo significant changes throughout life or in response to external factors, there are epigenetic modifications such as DNA methylation that affect the activity of genes and their transcription. Third, because the genetic sequence itself does not change with the development of most diseases (clearly with the exception of most tumours), somatic genomics is of limited use in mapping the precise status of cardiovascular diseases such as hypertension. The main role of genomic data lies in risk prediction.

We do not discuss the important aspect of pharmacogenomics in this article because it is covered in detail in another article in this series.^18^ However, it is clear that genomics offers data for all facets of research and clinical practice, from dissection of individual pathophysiological principles to prediction of the risk of hypertension developing and its related complications, as well as to determination of therapeutic response.

## Transcriptomics

There are few transcriptomic studies in hypertension. The reasons for this are probably manifold. First, and this also applies to the other omics technologies discussed further on, the transcriptome is much more cell and tissue specific than is the genome, and the choice of sample matters. Second, the complexity of data is even higher than that of the genome; focusing only on genes, there can be multiple transcripts of the same gene and the importance of intergenic regulatory sections of DNA are incompletely understood. Third, minute changes in the regulation of a specific gene can have much more dramatic downstream effects on translation to protein and protein or enzyme activity. Currently applied statistical techniques that focus on fold changes of expression of individual genes are not able to capture the complexities of regulatory networks. Fourth, even if technology for transcriptome-wide screening in the form of microarrays is available, validation of findings with an independent technique such as real-time quantitative polymerase chain reaction is required, which adds to the complexity and costs of the experiment. Finally, RNA is far less stable than DNA, and consistent sample handling and processing are crucial for the generation of high-quality data. All these issues limit the use of transcriptomics to dedicated questions in a research environment. Needless to say, however, the transcriptome should be able to provide much needed information about how the general potential of the genome is activated and transcribed in a tissue-specific manner and thereby deliver a unique fingerprint of an individual's molecular makeup.

A number of research studies have used gene expression profiling to study patients with hypertension. A study by Marques et al.^19^ in renal tissue of patients with hypertension and normotensive controls highlighted 14 genes that were differentially expressed in the medulla. Of the genes that were confirmed in further experiments, *REN*, encoding renin, and *CD36*, encoding CD36, provided important functional insights. The latter is particularly interesting because a study by Korkor et al.^20^ in peripheral blood, a material much more easily obtained in patients with hypertension, also found regulation of expression of genes involved in major histocompatibility complex class II receptor activity and immune response. Another study in peripheral blood by Stoynev et al.^21^ compared gene expression profiles between patients with hypertension, those with type 2 diabetes, and controls and found *PDGFRB*, encoding platelet-derived growth factor beta, significantly regulated. There were a number of other genes encoding adhesion molecules and growth factors that were found to be differentially expressed, but the results should be assessed critically in view of the selected array that focused on 84 targets and did not provide genome-wide coverage.

In hypertensive disorders other than essential hypertension, there may be lower-hanging fruit with regard to gene expression studies. In secondary hypertension, in which the main driving force of elevated blood pressure is known, gene expression as well as next-generation sequencing studies may play important roles. For example, in primary hyperaldosteronism, such studies can unravel the mechanisms of autonomous aldosterone production, as reviewed recently by Monticone et al.^22^ In rapidly developing hypertensive conditions such as pre-eclampsia, studies can be performed at different time points to explore changes in regulation and also with regard to prediction of the development of these conditions. A number of such studies in placental tissue and in peripheral blood have been performed and highlight differential regulation of genes, many of which are involved in immune mechanisms.^23^, ^24^, ^25^

Studies into mRNA expression are subject to limited RNA stability and tissue-specific gene expression, and these issues also apply to studies of microRNA expression, albeit to a lesser extent. MicroRNAs are master regulators and can control the expression of a large number of genes. The same applies to other noncoding RNAs that may provide important insights into the pathogenesis of hypertension.^26^ MicroRNAs are more stable in biological samples and can be extracted from blood and urine; there is general consensus that peripheral profiles from these substances provide at least some meaningful information about organ-specific expression.^27^ For example, a study by Parthenakis et al.^28^ looked into microRNA profiles in peripheral blood cells from patients with hypertension and microalbuminuria. Even if this was a targeted study into a limited number of microRNAs, it gives an indication of the potential impact that an omics approach could have. The true value of microRNA profiling, however, can be seen when it is used together with mRNA expression data. In fact, the previously mentioned study by Marques et al.^19^ benefited from such data; they not only showed differential *REN* expression but also that miR-663 and miR-181a contributed to its regulation. We have recently shown differential expression of miR-206 in the development of pre-eclampsia and a link to expression of *IGF1* encoding insulin-like growth factor 1.^29^

## Proteomics

We previously reviewed the potential of proteomics in the field of hypertension in detail.^30^, ^31^ Proteomics shares many challenges with transcriptomics and metabolomics.

1. The proteome varies between tissues, and different results will be obtained depending on the choice of substrate for analysis.
2. Appropriate and standardized preparation of samples is essential to enable comparability between studies.
3. The wide concentration range of proteins in biological samples requires high-sensitivity platforms to detect proteins and peptides in the lowest concentration range, and masking of low-concentration proteins by more abundant proteins (eg, albumin in plasma samples) can be problematic.
4. Depending on the analysis platform, different results can be obtained. This relates to the concentration range and molecular weight of proteins that can be detected with a given platform and the biostatistical prediction/identification of source proteins from polypeptides in a sample. A recent American Heart Association (AHA) statement is enthusiastic about the potential of proteomics in cardiovascular diseases but calls for standardization of protocols and large-scale funding initiatives to catch up, particularly with genomics.^7^

A few recent examples highlight the potential of proteomics to identify proteins involved in the pathogenesis of hypertension. For example, the urinary proteomic study by Matafora et al.^32^ described different levels of uromodulin depending on the *UMOD* genotype and also found nephrin-1 to be associated with salt-sensitive hypertension. However, more work has been done in the prediction and diagnosis of complications of hypertension. Kuznetsova et al.^33^ used urinary proteomics to develop a panel of peptides that are characteristic for early diastolic dysfunction in patients with hypertension. Peptides within this panel were also differentially expressed in patients with overt heart failure and controls, indicating that molecular processes involved in early stages of organ damage still play a role in advanced disease. A recent plasma proteomic study by Pena et al.^34^ in hypertensive patients and in patients with diabetes identified biomarker signatures characteristic for transition in albuminuria status, which could help to identify renal complications of hypertension at an early stage. In a case-control study nested within the **A**nglo-**S**candinavian **C**ardiac **O**utcomes **T**rial (ASCOT) cohort, we demonstrated that urinary proteomic signatures can predict cardiovascular events in hypertensive patients.^35^

Proteomics is an attractive technology to study specific hypertensive conditions such as pre-eclampsia. The opportunities lie in the rapid development of the condition, the possibility of taking serial samples along its development, and of course the clinical need resulting from the morbidity and mortality that are associated with pre-eclampsia. A relatively large number of studies have therefore looked into proteomic markers of pre-eclampsia.^36^, ^37^ Many published studies are, however, characterized by small sample size and lack of external validation or functional dissection of findings. A noteworthy exception is a study by Myers et al.,^38^ who combined unbiased screening with targeted validation in independent discovery and validation cohorts to build various predictive biomarker panels. However, even this relatively large and externally validated study would not be able to “compete” with genetic and genomic studies in this field and generally in hypertension, indicating again the complexity of and lack of large-scale funding for proteomic studies.

## Metabolomics

In metabolomic research, we also see the use of different platforms, some of which are mass spectrometry–based, as are many contemporary proteomic approaches; others are based on nuclear magnetic resonance (NMR) spectroscopy or on arrays specific for a range of metabolites. Targeted and nontargeted approaches and the use of different platforms that all cover only a certain range of metabolites can make it difficult to compare studies and their results. The metabolome is driven by the availability of substrates and enzyme activity and is thereby a few steps further away from the genome than, eg, the transcriptome and the proteome. It is therefore particularly well suited to assess environmental influences and the current state of the organism, not least because the metabolome adapts quickly to physiological and pathophysiological conditions.

Few studies have used metabolomic techniques to identify metabolites causally linked to the pathogenesis of hypertension. One recent study identified the dicarboxylic acid hexadecanedioate to be associated with hypertension and mortality and demonstrated that oral hexadecanedioate increased blood pressure and vascular response to noradrenaline in Wistar-Kyoto rats.^39^ Another recent study identified a number of metabolites associated with a higher risk (serine, glycine, and acyl-alkyl-phosphatidylcholines C42:4 and C44:3) or a lower risk (diacyl-phosphatidylcholines C38:4 and C38:3) of incident hypertension over a 10-year period^40^ This study does not benefit from external validation, and functional dissection of the results has not yet taken place. Nevertheless, the idea that metabolic changes precede the development of hypertension is in keeping with both our concept of the pathophysiology of the condition and the interaction between genetic and environmental factors, rendering the data appealing. Regarding the prediction of target-organ damage, in an NMR spectroscopy-based metabolic study, Zhang et al.^41^ found associations between echocardiographic indices of left ventricular diastolic function and a range of circulating metabolites involved in energy substrate use and protection against oxidative stress.

The greatest potential of metabolomic studies in hypertension may, however, lie in the monitoring of treatment responses. Rotroff et al.^42^ demonstrated that metabolite profiles change in response to treatment with hydrochlorothiazide, differ between ethnicities, and are able to predict treatment success. The same group of authors also used these data together with genomic data in an integrative analysis to identify genetic markers that predict response to hydrochlorothiazide in a study by Shahin et al.^43^ Importantly, the recently developed assays for comprehensive monitoring of drug metabolites in urine use metabolomic approaches and have already been introduced into clinical practice to assess adherence to therapy.^44^

## Interactome and Systems Biology

Following the molecular biology paradigm from gene to protein and metabolite, it appears reasonable to assume that genomic, transcriptomic, proteomic, and metabolomic studies detail the same phenomena from different angles. Combining the information from different omics technologies should provide a more holistic view of processes involved in physiology and pathophysiology. This integrative approach that describes the interactome is the basis of systems biology. There are currently no truly comprehensive interactome data in human hypertension available. However, the tools to bring complex data together are being developed, and some examples show the direction in which this research is heading. In a study by Atanur et al.^45^ in rat models of hypertension that underwent extensive genotyping and phenotyping, loci for strain-specific disease processes and loci that overlap between strains as well as with loci for human traits have been identified. The biostatistical and bioinformatic tools used to analyze this extremely complex data set can inform similar analyses in human data sets. Other rodent studies used systems biology approaches to place proteomic data into larger disease pathways, identifying dysregulated networks and predicting further targets from such analyses.^46^, ^47^ In human hypertension, 2 recent studies by Marrachelli et al.^48^, ^49^ combined metabolomic and genomic data to study factors associated with microalbuminuria and a range of cardiometabolic risk factors. The previously mentioned article by Shahin et al.^43^ is another example of the integration of genetic and metabolomic data.

The potential of systems biology approaches to study the pathophysiology of hypertension and other vascular diseases is evident.^50^ Integrating data from different omics approaches may even help to overcome some of the limitations of individual technologies by looking at a broader picture of disease networks rather than at specific compounds in isolation.

## Future Role of Omics in Hypertension: Focus on Clinical Applications

Predicting the future of omics in hypertension is of course impossible, but we can draw a picture that is based on past and present achievements and the unmet needs in research and clinical practice. It is clear that omics technologies that are already far advanced and have generated large amounts of data, in particular genomics, are more likely to continue playing a major role in the years to come when compared with other technologies that are still in their infancy. In keeping with the maturity of genomics research, recent statements by the AHA therefore outline very precisely the future use of genomics for translational and clinical implementation.^51^, ^52^ However, the huge potential impact of proteomics and other omics technologies in cardiovascular research and clinical practice has also been acknowledged by the AHA.^7^ We highlight a few areas in hypertension that will most likely benefit from omics technologies in the near future.

### Diagnosis of hypertension

It is clear that the diagnosis of hypertension will continue to be based on blood pressure measurements; there is no need for complex omics tests to replace a simple diagnostic procedure. However, omics data can provide detailed insight into the makeup of hypertension in individual patients and therefore supplement established diagnostic procedures to direct therapies.

When it comes to clinical applications, we see in the first instance a role for genomic studies. The genome remains virtually static throughout life and always defines a potential or risk, whereas the transcriptome, proteome, and metabolome not only change throughout life but also change in response to disease processes, making it more difficult to differentiate between cause and consequence. Currently, genetic techniques are already applied in the diagnosis of rare forms of hypertension or to classify subtypes of adrenal tumours; in the latter case there can be organ-specific mutations that would not be detectable in a somatic genetic screen, which would probably be most useful in patients with essential hypertension. Other omics technologies are important in a research setting but may not find their way into clinical practice in the very near future.

### Prediction of hypertension

Identifying patients at risk at an early stage of the disease process is a generally attractive concept. With deeper understanding of the genetic determinants, the prediction of hypertension later in life is already possible to some extent using genetic risk scores.^53^ The genetic background of an individual is the prime driver of risk but will be modified by the environment, which forms the basis for preventive strategies such as lifestyle modifications to prevent or delay the onset of hypertension. It can be expected that early and subclinical stages of hypertension will be characterized by alterations in gene and protein expression. Detection of altered profiles using transcriptomic, proteomic, and metabolomic techniques could provide information about which patients are on a trajectory toward overt hypertension. We are not in a position to discuss whether prediction of hypertension would indeed have a traceable effect on clinical outcomes. The few studies in prehypertension, such as the **Tr**ial of **P**reventing **Hy**pertension (TROPHY), showed that the onset of overt hypertension can be prevented or at least delayed in some patients.^54^ It is possible, however, that interventions would be even more successful if they came earlier and at a stage at which the molecular alterations toward hypertension are less advanced.

### Prediction of complications

Hypertension is also a risk factor for complications such as heart failure, stroke, and renal failure. Early detection of such complications and targeted preventive measures remain the holy grail in the management of patients with hypertension. Current clinical practice is centred on blood pressure values and simple clinical markers of organ damage such as albuminuria and left ventricular hypertrophy. More sophisticated cardiovascular phenotyping has been suggested to help target therapies to those at greatest risk.^55^

Omics-based signatures may add another aspect to the assessment of target-organ damage. There are already clinical studies providing evidence that proteomic signatures can be reliably detected and linked to subclinical organ damage in cardiac and renal diseases.^56^, ^57^ Such signatures, also taking other omics technologies into account, could be further developed to obtain a global view of cardiovascular damage in patients with hypertension. Time will tell whether such assessment is feasible and can meaningfully impact treatment decisions.

### Stratified clinical trials and targeting of treatment

Before omics-derived data can be translated into widespread clinical use, it must be demonstrated that they can reliably predict outcomes and inform treatment decisions. This can be done in the form of stratified clinical trials in which inclusion into the trial or treatment allocation, or both, are determined by omics signatures. In fact, this concept is attractive for funding bodies, researchers, and study participants, because only those who are at greatest risk will be exposed to investigational medicines. A trial in patients with type 2 diabetes at risk of diabetic nephropathy based on proteomic signatures is currently under way,^58^ and a recently formed consortium is studying the use of omics signatures to predict the clinical course of primary hyperaldosteronism with the aim of precisely targeting treatment for these patients (http://www.ensat-ht.eu). There are also some attempts to use specific results from omics screens to inform clinical practice. For example, variants of the *UMOD* gene that was discovered to be associated with hypertension in a GWAS have the potential to predict the antihypertensive response to loop diuretics,^12^ and a clinical trial is currently planned (British Heart Foundation Clinical Study CS/16/1/31878).

### Monitoring of treatment response

In patients with hypertension, treatment response can be monitored by changes in blood pressure, and in turn the change in blood pressure modifies cardiovascular risk. However, although the relationship between blood pressure and cardiovascular outcome is tight at a population level, it does not necessarily apply to individual patients; in this case, additional markers of treatment response could help in clinical management.

Clearly, genetic markers will be of limited use in this respect because they are not sufficiently dynamic, but epigenetic, transcriptomic, proteomic, and metabolomic markers could well be suited to assess treatment response. Longer-term changes in proteomic signatures in response to antihypertensive treatment with the angiotensin-receptor blocker irbesartan have already been shown,^56^ and we referred earlier to changes in the metabolome in response to hydrochlorothiazide.^42^

### A molecular definition of hypertension

In this section, we have focused on the potential for future clinical application of omics technologies in hypertension. Precise diagnosis, prediction of hypertension, prediction of complications, and targeting of treatment are all cornerstones of precision medicine and of clinical relevance. There is the additional aspect of a better understanding of pathophysiology that will almost automatically accompany these clinical applications and complement basic and clinical research into the condition. In other areas, such as pancreatic cancer, we have witnessed how a deep understanding of the molecular makeup can help to redefine the disease.^59^ There is no reason why comprehensive molecular characterization of hypertension should not be able to define disease subtypes and lead us away from a merely blood pressure–based definition of hypertension in the future.

## Challenges on the Way Ahead

In this review, we have tried to outline some of the potential of omics technologies in future clinical and research applications in hypertension. We are convinced that this potential is huge but accept that omics studies in hypertension are not without challenges.

Targeted assessment of genetic variants, protein biomarkers, and metabolites is without doubt important in the diagnosis and management of some patients, in particular patients with secondary forms of hypertension. In contrast, primary (essential) hypertension is a multifactorial condition in which there is little difference in the overall genetic makeup, even between patients at the extremes of the blood pressure distribution.^3^ There is currently no evidence that comprehensive assessment of large numbers of genetic variants, proteins, and metabolites provides information beyond investigations that form the basis of contemporary clinical practice in these patients. One reason for the lack of translation to the clinic is the complexity of omics data. Results are difficult to interpret and to contextualize clinically in individual patients. The other reason is related to the costs of omics studies. With advancing technology, we have witnessed cost reductions, especially in the areas of genomics and exome sequencing. The National Human Research Institute states that “the cost to generate a high-quality 'draft' whole human genome sequence in mid-2015 was just above $4,000; by late in 2015, that figure had fallen below $1,500. The cost to generate a whole-exome sequence was generally below $1,000” but acknowledges that it is difficult to provide estimates for these figures (https://www.genome.gov/27565109/the-cost-of-sequencing-a-human-genome). In the areas of proteomics and metabolomics, costs are even more difficult to estimate because of the wide range of available technologies. It should be noted that in most clinical studies, targeted approaches to assess proteins and metabolites have been applied; these may be relatively inexpensive but can provide information on only limited numbers of molecules and are not truly comprehensive omics approaches. There is a body of literature that assesses the cost-effectiveness of biomarker screening before the initiation of primary cardiovascular disease interventions, as discussed in a systematic review produced by the Belgian Health Care Knowledge Centre.^60^ These studies are not necessarily of the highest scientific quality, and in the Belgian review, only 1 article on the value of high-sensitivity C-reactive protein testing in cardiovascular disease–free individuals compared this biomarker with traditional risk scoring.^61^ Detailed health economic analyses into novel biomarkers including data derived from omics studies are currently not available in cardiovascular diseases in general or in hypertension in particular.

We have focused our review on studies in humans but appreciate that in light of the already available wealth of omics studies, it is now important to further dissect these findings and understand the pathophysiology and potential therapeutic benefit related to these data. Studies in well-established animal models (spontaneously hypertensive rat, deoxycorticosterone acetate renovascular hypertension) could demonstrate that the omics approach accrues new additional data, could establish the tools to analyze and integrate such data, and in fact could demonstrate that “interactome” analysis is possible and provides meaningful information. Some of this work has already been achieved in rodent models.^45^, ^62^ Another underexplored area is the study of omics data in mendelian and other forms of secondary hypertension in which key pathophysiological pathways are known and the value of additional insights can be judged against existing knowledge. Such studies are currently under way in primary aldosteronism (www.ensat-ht.eu).

Against this background, it is important to focus on key steps toward the implementation of omics technologies in hypertension. The first is to raise awareness of the global burden of hypertension and the need for more precise diagnosis and treatment. The second is to exploit existing omics data by translating key findings, especially from GWAS, into mechanistic and clinical studies; in light of the high costs of omics research, it will be difficult without such clinical success stories to “compete” with other areas in medicine. The third is to not only focus on the molecular aspects of hypertension but also to improve on the clinical characterization of patients, eg, by using ambulatory blood pressure data as the basis for omics studies as opposed to nonstandardized office blood pressure readings. Finally, we must standardize and streamline research in transcriptomics, proteomics, and metabolomics in the same way that genomic consortia have been established in recent years to pool data sets and financial resources of a size and quality never before seen in hypertension research.

## Funding Sources

This work has been supported by a Centre of Research Excellence grant from the British Heart Foundation (RE/13/5/30177) and by grants from the European Commission (“sysVASC”, Grant Agreement 603288; “PRIORITY”, Grant Agreement 279277; and “HOMAGE”, Grant Agreement 305507).

## Disclosures

The authors have no conflicts of interest to disclose.

## Figures and Tables

**Table 1:** Challenges related to omics technologies

Challenge	Specific issues
All omics technologies involve the generation of large amounts of data	Data processing, analysis, and interpretation is complicated
	Statistical tools that help decide which of the many features that were analyzed are biologically relevant have been developed but remain suboptimal
	Extensive replication in independent cohorts and validation and functional dissection using other methods remain the cornerstones of scientific work in the omics era
The complexity of data depends on the depth of screening and the particular omics topic	Single-nucleotide polymorphism–based, genome-wide association study vs exome or whole genome sequencing
	Single genes can have multiple transcripts that translate the same gene into different proteins that can themselves be post-translationally modified
	As a general rule, the complexity of features is thought to increase in the cascade from genomics to transcriptomics and proteomics and probably metabolomics
Precision and coverage of technologies vary across the omics topics	By default, at least at the single DNA base pair level, there are only 4 possible features (adenine, cytosine, guanine, thymine), and with current genotyping technologies these can be assessed precisely and with virtually 100% sensitivity and specificity
	In contrast, proteomic and metabolomic technologies are less sensitive and specific; because of technical restrictions, no single technology can detect all possible features, and all these features cannot be identified precisely

## References

[1] Olsen M.H., Angell S.Y., Asma S. (2016). A call to action and a lifecourse strategy to address the global burden of raised blood pressure on current and future generations: the Lancet Commission on hypertension. Lancet.

[2] Delles C., Padmanabhan S. (2012). Genetics and hypertension: is it time to change my practice?. Can J Cardiol.

[3] Padmanabhan S., Newton-Cheh C., Dominiczak A.F. (2012). Genetic basis of blood pressure and hypertension. Trends Genet.

[4] Davis B.D. (1949). The Isolation of biochemically deficient mutants of bacteria by means of penicillin. Proc Natl Acad Sci U S A.

[5] Kaplan N.M. (1998). Commentary on the sixth report of the Joint National Committee (JNC-6). Am J Hypertens.

[6] Ehret G.B., Ferreira T., Chasman D.I. (2016). The genetics of blood pressure regulation and its target organs from association studies in 342,415 individuals. Nat Genet.

[7] Lindsey M.L., Mayr M., Gomes A.V. (2015). transformative impact of proteomics on cardiovascular health and disease: A scientific statement from the American Heart Association. Circulation.

[8] Caulfield M., Munroe P., Pembroke J. (2003). Genome-wide mapping of human loci for essential hypertension. Lancet.

[9] Wellcome Trust Case Control Consortium (2007). Genome-wide association study of 14,000 cases of seven common diseases and 3,000 shared controls. Nature.

[10] Ehret G.B., Munroe P.B., Rice K.M., International Consortium for Blood Pressure Genome-Wide Association S (2011). Genetic variants in novel pathways influence blood pressure and cardiovascular disease risk. Nature.

[11] Padmanabhan S., Caulfield M., Dominiczak A.F. (2015). Genetic and molecular aspects of hypertension. Circ Res.

[12] Padmanabhan S., Melander O., Johnson T. (2010). Genome-wide association study of blood pressure extremes identifies variant near UMOD associated with hypertension. PLoS Genet.

[13] Surendran P., Drenos F., Young R. (2016). Trans-ancestry meta-analyses identify rare and common variants associated with blood pressure and hypertension. Nat Genet.

[14] Ji W., Foo J.N., O'Roak B.J. (2008). Rare independent mutations in renal salt handling genes contribute to blood pressure variation. Nat Genet.

[15] Scholl U.I., Nelson-Williams C., Yue P. (2012). Hypertension with or without adrenal hyperplasia due to different inherited mutations in the potassium channel KCNJ5. Proc Natl Acad Sci U S A.

[16] Scholl U.I., Goh G., Stolting G. (2013). Somatic and germline CACNA1D calcium channel mutations in aldosterone-producing adenomas and primary aldosteronism. Nat Genet.

[17] Parmar P.G., Taal H.R., Timpson N.J. (2016). International genome-wide association study consortium identifies novel loci associated with blood pressure in children and adolescents. Circ Cardiovasc Genet.

[18] Johnson JA. Whole transcriptome profiling: an RNA seq primer and implications for hypertension genomics and pharmacogenomics research. Can J Cardiol [in press]. 10.1111/cts.12511 PMC5866981 28945944

[19] Marques F.Z., Campain A.E., Tomaszewski M. (2011). Gene expression profiling reveals renin mRNA overexpression in human hypertensive kidneys and a role for microRNAs. Hypertension.

[20] Korkor M.T., Meng F.B., Xing S.Y. (2011). Microarray analysis of differential gene expression profile in peripheral blood cells of patients with human essential hypertension. Int J Med Sci.

[21] Stoynev N., Dimova I., Rukova B. (2014). Gene expression in peripheral blood of patients with hypertension and patients with type 2 diabetes. J Cardiovasc Med (Hagerstown).

[22] Monticone S., Else T., Mulatero P., Williams T.A., Rainey W.E. (2015). Understanding primary aldosteronism: impact of next generation sequencing and expression profiling. Mol Cell Endocrinol.

[23] Leavey K., Benton S.J., Grynspan D. (2016). unsupervised placental gene expression profiling identifies clinically relevant subclasses of human preeclampsia. Hypertension.

[24] Textoris J., Ivorra D., Ben Amara A. (2013). Evaluation of current and new biomarkers in severe preeclampsia: a microarray approach reveals the VSIG4 gene as a potential blood biomarker. PLoS One.

[25] Rajakumar A., Chu T., Handley D.E. (2011). Maternal gene expression profiling during pregnancy and preeclampsia in human peripheral blood mononuclear cells. Placenta.

[26] Romaine S.P., Charchar F.J., Samani N.J., Tomaszewski M. (2016). Circulating microRNAs and hypertension—from new insights into blood pressure regulation to biomarkers of cardiovascular risk. Curr Opin Pharmacol.

[27] Creemers E.E., Tijsen A.J., Pinto Y.M. (2012). Circulating microRNAs: novel biomarkers and extracellular communicators in cardiovascular disease?. Circ Res.

[28] Parthenakis F.I., Marketou M.E., Kontaraki J.E. (2016). Comparative microRNA profiling in relation to urinary albumin excretion in newly diagnosed hypertensive patients. J Hum Hypertens.

[29] Akehurst C., Small H.Y., Sharafetdinova L. (2015). Differential expression of microRNA-206 and its target genes in preeclampsia. J Hypertens.

[30] Carty D.M., Schiffer E., Delles C. (2013). Proteomics in hypertension. J Hum Hypertens.

[31] Delles C., Neisius U., Carty D.M. (2012). Proteomics in hypertension and other cardiovascular diseases. Ann Med.

[32] Matafora V., Zagato L., Ferrandi M. (2014). Quantitative proteomics reveals novel therapeutic and diagnostic markers in hypertension. BBA Clin.

[33] Kuznetsova T., Mischak H., Mullen W., Staessen J.A. (2012). Urinary proteome analysis in hypertensive patients with left ventricular diastolic dysfunction. Eur Heart J.

[34] Pena M.J., Jankowski J., Heinze G. (2015). Plasma proteomics classifiers improve risk prediction for renal disease in patients with hypertension or type 2 diabetes. J Hypertens.

[35] Brown C.E., McCarthy N.S., Hughes A.D. (2015). Urinary proteomic biomarkers to predict cardiovascular events. Proteomics Clin Appl.

[36] Kolialexi A., Mavreli D., Tounta G., Mavrou A., Papantoniou N. (2015). Urine proteomic studies in preeclampsia. Proteomics Clin Appl.

[37] Klein J., Buffin-Meyer B., Mullen W. (2014). Clinical proteomics in obstetrics and neonatology. Expert Rev Proteomics.

[38] Myers J.E., Tuytten R., Thomas G. (2013). Integrated proteomics pipeline yields novel biomarkers for predicting preeclampsia. Hypertension.

[39] Menni C., Graham D., Kastenmuller G. (2015). Metabolomic identification of a novel pathway of blood pressure regulation involving hexadecanedioate. Hypertension.

[40] Dietrich S., Floegel A., Weikert C. (2016). Identification of serum metabolites associated with incident hypertension in the European prospective investigation into cancer and nutrition-potsdam study. Hypertension.

[41] Zhang Z.Y., Marrachelli V.G., Thijs L. (2016). Diastolic left ventricular function in relation to circulating metabolic biomarkers in a general population. J Am Heart Assoc.

[42] Rotroff D.M., Shahin M.H., Gurley S.B. (2015). Pharmacometabolomic assessments of atenolol and hydrochlorothiazide treatment reveal novel drug response phenotypes. CPT Pharmacometrics Syst Pharmacol.

[43] Shahin M.H., Gong Y., McDonough C.W. (2016). A genetic response score for hydrochlorothiazide use: insights from genomics and metabolomics integration. Hypertension.

[44] Tomaszewski M., White C., Patel P. (2014). High rates of non-adherence to antihypertensive treatment revealed by high-performance liquid chromatography-tandem mass spectrometry (HP LC-MS/MS) urine analysis. Heart.

[45] Atanur S.S., Diaz A.G., Maratou K. (2013). Genome sequencing reveals loci under artificial selection that underlie disease phenotypes in the laboratory rat. Cell.

[46] Husi H., Van Agtmael T., Mullen W. (2014). Proteome-based systems biology analysis of the diabetic mouse aorta reveals major changes in fatty acid biosynthesis as potential hallmark in diabetes mellitus-associated vascular disease. Circ Cardiovasc Genet.

[47] Husi H., Sanchez-Nino M.D., Delles C. (2013). A combinatorial approach of proteomics and systems biology in unravelling the mechanisms of acute kidney injury (AKI): involvement of NMDA receptor GRIN1 in murine AKI. BMC Syst Biol.

[48] Marrachelli V.G., Monleon D., Rentero P. (2014). Genomic and metabolomic profile associated to microalbuminuria. PLoS One.

[49] Marrachelli V.G., Rentero P., Mansego M.L. (2016). Genomic and metabolomic profile associated to clustering of cardio-metabolic risk factors. PLoS One.

[50] Dominiczak A.F., Herget-Rosenthal S., Delles C. (2010). Systems biology to battle vascular disease. Nephrol Dial Transplant.

[51] Fox C.S., Hall J.L., Arnett D.K. (2015). Future translational applications from the contemporary genomics era: a scientific statement from the American Heart Association. Circulation.

[52] Musunuru K., Hickey K.T., Al-Khatib S.M. (2015). Basic concepts and potential applications of genetics and genomics for cardiovascular and stroke clinicians: a scientific statement from the American Heart Association. Circ Cardiovasc Genet.

[53] Smith J.A., Ware E.B., Middha P., Beacher L., Kardia S.L. (2015). Current Applications of genetic risk scores to cardiovascular outcomes and subclinical phenotypes. Curr Epidemiol Rep.

[54] Julius S., Nesbitt S.D., Egan B.M. (2006). Feasibility of treating prehypertension with an angiotensin-receptor blocker. N Engl J Med.

[55] Mancia G., Fagard R., Narkiewicz K. (2013). 2013 ESH/ESC guidelines for the management of arterial hypertension: the task force for the management of arterial hypertension of the European Society of Hypertension (ESH) and of the European Society of Cardiology (ESC). J Hypertens.

[56] Delles C., Schiffer E., von Zur Muhlen C. (2010). Urinary proteomic diagnosis of coronary artery disease: identification and clinical validation in 623 individuals. J Hypertens.

[57] Schanstra J.P., Zurbig P., Alkhalaf A. (2015). Diagnosis and prediction of CKD progression by assessment of urinary peptides. J Am Soc Nephrol.

[58] Lindhardt M., Persson F., Currie G. (2016). Proteomic prediction and renin angiotensin aldosterone system inhibition prevention of early diabetic nephropathy in type 2 diabetic patients with normoalbuminuria (PRIORITY): essential study design and rationale of a randomised clinical multicentre trial. BMJ Open.

[59] Bailey P., Chang D.K., Nones K. (2016). Genomic analyses identify molecular subtypes of pancreatic cancer. Nature.

[60] Roberfroid D, San Miguel L, Thiry N. Novel serum biomarkers for the prediction of cardiovascular risk. Health Technology Assessment (HTA) Brussels: Belgian Health Care Knowledge Centre (KCE), 2013. KCE Reports 201. D/2013/10.273/22. Available at: https://kce.fgov.be/sites/default/files/page_documents/KCE_201_Novel_serum_biomarkers_for_the_prediction_of_cardiovascular_risk.pdf. Accessed December 30, 2016.

[61] Lee K.K., Cipriano L.E., Owens D.K., Go A.S., Hlatky M.A. (2010). Cost-effectiveness of using high-sensitivity C-reactive protein to identify intermediate- and low-cardiovascular-risk individuals for statin therapy. Circulation.

[62] Low T.Y., van Heesch S., van den Toorn H. (2013). Quantitative and qualitative proteome characteristics extracted from in-depth integrated genomics and proteomics analysis. Cell Rep.

